# Supplementary material for: The Systems Biology Research Tool: evolvable open-source software
Source: BMC Syst Biol. 2008 Jun 29;2:55. doi: 10.1186/1752-0509-2-55 (PMC2446383; doi:10.1186/1752-0509-2-55)
Supplement: Additional file 1 — SBRT Archive. An archive of the current version of the Systems Biology Research Tool. [file 1752-0509-2-55-S1.zip › sbrt-1.4.0/doc/developers_guide/api/sbrt/kernel/math/optimization/class-use/ProgramSolver.html]

Uses of Interface sbrt.kernel.math.optimization.ProgramSolver


|  |  |  |
| --- | --- | --- |
| **Overview** \| **Package** \| **Class** \| **Use** \| **Tree** \| **Deprecated** \| **Index** \| **Help** | |  |
| PREV   NEXT | **FRAMES**    **NO FRAMES**     **All Classes** |  |


---


## **Uses of Interface sbrt.kernel.math.optimization.ProgramSolver**

| Packages that use ProgramSolver | |
| --- | --- |
| **sbrt.ext.cplex** | Provides a class that allows communication with CPLEX. |
| **sbrt.ext.glpk** | Provides a class that allows communication with GLPK. |
| **sbrt.kernel.fba.comp** | Provides classes and interfaces for performing Flux Balance Analysis computations. |
| **sbrt.kernel.math.optimization** | Provides classes and interfaces for creating objects relevant to solving optimization problems. |
| **sbrt.shell** | Provides classes used by shell-level software. |

Uses of ProgramSolver in sbrt.ext.cplex

| Classes in sbrt.ext.cplex that implement ProgramSolver | |
| --- | --- |
| `class` | `CplexSolver<R,C>`             This class is a wrapper for the CPLEX package from ILOG. |

Uses of ProgramSolver in sbrt.ext.glpk

| Classes in sbrt.ext.glpk that implement ProgramSolver | |
| --- | --- |
| `class` | `GlpkSolver<R,C>`             This class is a wrapper for the GLPK package from GNU. |

Uses of ProgramSolver in sbrt.kernel.fba.comp

Constructors in sbrt.kernel.fba.comp with parameters of type ProgramSolver

`FbaOptComputer(IrrevRxnFluxome fluxome,
ProgramSolver<java.lang.Object,java.lang.Object> solver)`
  
          Constructs a new FBA optimization computer from the
provided fluxome and program solver.

`FbaOptComputer(IrrevRxnFluxome fluxome,
ProgramSolver<java.lang.Object,java.lang.Object> solver,
double constraintTolerance)`
  
          Constructs a new FBA optimization computer from the
provided fluxome, program solver, and constraint tolerance.

`MsFluxomeReducer(ProgramSolver<java.lang.Object,java.lang.Object> solver)`
  
          Constructs a fluxome reducer from the provided linear program solver.

`WwFluxomeReducer(ProgramSolver<java.lang.Object,java.lang.Object> solver)`
  
          Constructs a fluxome reducer from the provided linear
program solver.

Uses of ProgramSolver in sbrt.kernel.math.optimization

| Subinterfaces of ProgramSolver in sbrt.kernel.math.optimization | |
| --- | --- |
| `interface` | `LinearProgramSolver<R,C>`             This interface is used to represent linear program solvers. |
| `interface` | `QuadraticProgramSolver<R,C>`             This interface is used to represent quadratic program solvers. |

Uses of ProgramSolver in sbrt.shell

Methods in sbrt.shell that return ProgramSolver

`static

<R,C> ProgramSolver<R,C>`

`ProgramSolverFactory.make(ManagerInput<java.lang.String,java.lang.String> input,
java.io.PrintWriter out)`
  
          Uses the information contained in the provided input to create a new
program solver object.

---


|  |  |  |
| --- | --- | --- |
| **Overview** \| **Package** \| **Class** \| **Use** \| **Tree** \| **Deprecated** \| **Index** \| **Help** | |  |
| PREV   NEXT | **FRAMES**    **NO FRAMES**     **All Classes** |  |


---
